# Japanese Adult Day Service Nurses' Bathing Decisions for Persons Requiring Long‐Term Care: A Focused Ethnography

**DOI:** 10.1111/opn.70052

**Published:** 2025-12-14

**Authors:** Kanako Miyoshi, Keiko Mori

**Affiliations:** ^1^ Graduate School of Health Sciences, Okayama University Okayama Japan

**Keywords:** adult day service, clinical judgement, community, home care, multidisciplinary collaboration, nurses, persons requiring care

## Abstract

**Introduction:**

Adult day services in Japan operate under the Long‐Term Care Insurance Law, and care is provided mainly by caregivers. However, because doctors are often not on site, nurses manage the health of the person requiring long‐term care. Adult day services provide bathing and functional training; however, although Japanese‐style bathing relieves fatigue and brings a sense of well‐being, it also entails the risk of bathing accidents for those in need of care. To continue living at home, those in need of care who have difficulty bathing at home must be provided with safe bathing during adult day services and supported in returning home safely. Nurses are responsible for accurately assessing the health status of users and implementing safe bathing. This study aimed to identify how nurses working in adult day services make bathing decisions for home‐dwelling persons requiring long‐term care.

**Method:**

Qualitative manifest and latent content analyses were performed using a focused ethnography.

**Findings:**

Six themes were identified: ‘gather information to compare with baseline’, ‘make observations based on information from others to understand the big picture’, ‘give persons time to get in shape’, ‘consideration of life at home’, ‘determining the need for medical institutions’ and ‘devise ways to communicate to promote collaboration’.

**Conclusions:**

Adult day service nurses' decisions about whether to bathe persons requiring care are characterised by their emphasis on information from others, consideration of the home living conditions of persons requiring care and their wishes regarding bathing. In addition, based on their observations, they determine the need for cooperation with medical institutions and communicate this information to family members and multiple professions.

**Summary**

- What does this research add to existing knowledge in gerontology?
  ○ This ethnography provides practical knowledge for nurses working in the absence of a physician in adult day care services to ensure safe bathing and the continued well‐being of persons requiring long‐term care.
  ○ Nurses' decisions regarding adult day services, where there are few medical professionals, require them to focus on multidisciplinary situational awareness (including the emergence of concerns for any reason) and information from family members.
  ○ Adult day service nurses are responsible for making decisions and balancing risk with the needs of the person requiring long‐term care and the family's desire for bathing.
- What are the implications of this new knowledge for nursing care for and with older adults?
  ○ Understanding adult day service nurses' decisions will enable them to work in multiple disciplines to provide higher quality services.
  ○ For persons requiring long‐term care, especially those who are at risk of a change in condition, avoiding risk while providing bathing services in accordance with their wishes will contribute to their continued living at home.
- How could the findings be used to influence practice, education, research, and policy?
  ○ It shows that one of the roles of an adult day service nurse is to determine whether bathing is acceptable.
  ○ By establishing a foundation that consolidates information from multidisciplinary situational awareness and family members, adult day service nurses can facilitate decisions that are in the best interests of persons requiring long‐term care.

## 1 Introduction

Life expectancy worldwide is increasing and is projected to reach approximately 77.4 years by 2054 (United Nations 2024), heightening the demand for medical and nursing care Consequently, the number of convalescents living in communities with diseases and disabilities is increasing, and the importance of adult day services is increasingly being recognised as an alternative to institutional care for older adults with functional disabilities (Ellen et al. 2017; Gitlin et al. 2006; Park 2008).

In Japan, which has the world's highest proportion of older adults aged ≥ 65 years (29.3%) (Statistics Bureau of Japan 2025), a long‐term care insurance system was introduced in 2000 to accommodate the growing older adult population. Under this system, the need for nursing care is estimated based on the person's physical and mental condition and the use of medical treatment and classified into seven levels: ‘1–2 persons requiring support’ and ‘1–5 persons requiring long‐term care’. A person receiving care who has been certified as requiring long‐term care is called a ‘person requiring long‐term care’. Adult day services available to persons requiring long‐term care are covered by the long‐term public care insurance system, with co‐payments ranging from 10% to 30%, depending on income. Many facilities specialise in adult day services without overnight functions, but some residential facilities also provide adult day services. Typically, those in need of care are picked up from their homes in the morning and sent home in the evening. Adult care consists of bathing, meals, functional training, toilet care and recreation (Ministry of Health, Labour and Welfare 2025). The number of daily users varies from less than 18 to 60, depending on the size of the facility. Caregivers may be trained as caregivers, entry‐level workers or practitioners, or may be unqualified and seek certification while working. Unlicensed caregivers often perform support duties for trained caregivers, such as transportation, recreation, serving meals and cleaning. The facilities are primarily staffed by caregivers, with one or several nurses assigned to each facility. Because there are no doctors on site, nurses responsible for medical management are expected to make appropriate judgements about the health status of those requiring care (Mori et al. 2023).

In Japan, full‐body bathing, in which the body is washed and then soaked up to the shoulders in 40°C–42°C water, is common. Unlike in other countries where daily showering is preferred over bathing, for many Japanese people, daily bathing is not merely a means of maintaining cleanliness but also a cultural custom to cleanse oneself and relax (Traphagan 2004). In addition to warming the entire body, relaxing and relieving fatigue, soaking in hot water is thought to bring a sense of well‐being, improve sleep quality and alleviate symptoms of depression (Tochihara 2022). However, when older people bathe in a bathtub filled with hot water in a private bathroom, they are more likely to experience impaired consciousness and cardiac events, which can be fatal. The annual number of deaths due to bathing at home among older adults is projected to be 24,777 in 2025 and 27,337 in 2035 (Suzuki et al. 2017), and measures are being sought to accommodate the decline in physical function (Tai et al. 2024). Furthermore, a study on home‐visit bathing service provision for persons requiring long‐term care (Hayasaka et al. 2001) analysed 130 bathing accidents reported by 108 establishments. The results showed that 18 (14%), 46 (35%) and 62 (48%) accidents occurred before, during and after bathing, respectively, including four (3%) ‘other’ cases, 42 persons requiring long‐term care with impaired consciousness, 29 with pallor/cyanosis, 24 with hypotension, 12 with respiratory distress and 14 who died.

For those requiring long‐term care and whose physical functions have declined, it is important to maintain the cycle of providing functional training and bathing at adult day services and returning home to continue living at home.

Making sound decisions in uncertain situations is the foundation of quality nursing care and influences patient outcomes (Manetti 2019). Home‐visit bathing decisions have identified pain, medical treatment prior to bathing that could not be handled (cases in which it is difficult to manage the condition of ventilators, tracheostomies, gastrostomy sites or wounds prior to bathing) and a high level of care required as factors that influence caregivers to discontinue bathing (Taira et al. 2019). It has also been suggested that nurses in nursing homes, which are places of daily living, need to be aware of bathing not only for health management according to the degree of illness or disability but also from the perspective of daily living and for purposes other than cleanliness (Nakamura et al. 2005).

In Japanese adult day services, vital signs such as blood pressure and body temperature are measured before making the decision to bathe the person requiring long‐term care (Hayasaka et al. 2016). Additionally, adult day service nurses need to assess the physical functions of persons requiring care based on their experience and knowledge and make comprehensive judgements. However, how nurses interpret measurements such as blood pressure and body temperature, and how they make bathing decisions for persons requiring care, has not been adequately examined. Therefore, it is necessary to clarify nurses' perceptions of their decisions to bathe persons requiring care.

This study aimed to identify how nurses working in adult day services make bathing decisions for people requiring long‐term care who live at home. Clarifying the decision to bathe persons requiring long‐term care in adult day services could contribute to improving the quality of nursing care and promote multidisciplinary collaboration to support the continuation of home life for persons requiring long‐term care.

For this study, a person requiring long‐term care was defined as ‘a person certified by the Japanese long‐term care insurance system as requiring long‐term care in daily life’. Regarding judgement, based on Tanner's (Tanner 2006, 204) definition of clinical judgement, it refers to ‘an interpretation or conclusion about a patient's needs, concerns, or health problems and/or the decision to take action (or not), use or modify standard approaches or improvise new ones as deemed appropriate by the persons requiring long‐term care’.

## 2 Methods

Focused ethnography (Cruz and Higginbottom 2013; Roper and Shapira 2000; Wisnesky et al. 2022) was used for this study. Ethnography is a research method that aims to understand culture, such as patterns and perceptions of group behaviours and practices, based on what the researcher observes and the information obtained from the study participants. In this study, the judgement of nurses in the adult day service setting was considered a culture. Focused ethnography entails a shorter period of participant observation than traditional ethnography, which involves staying in the field for several years. The focused ethnography usually involves visiting the site multiple times over one to several days, consisting of short periods of one to 9 days per location (Kelly 2022). In this study, the first author (a nurse with a master's degree in education and a doctoral student in nursing at the time of the study) had worked in adult day care service for 4 years. Prior to the study, information was collected from websites and brochures of the target facilities under examination in order to learn about the characteristics of each facility unit, such as the daily flow and services provided, as well as to facilitate participant observation. This study conformed to Consolidated Criteria for Reporting Qualitative Research (COREQ) guidelines (Tong et al. 2007), an integrated standard for reporting qualitative research.

### 2.1 Setting and Participants

This study was conducted in Okayama City, Japan, between September 2023 and February 2024. To recruit participants, we first contacted the target facilities by telephone, explained the purpose of the study to the person responsible at the facility, and obtained verbal consent. I then visited the facility and obtained written consent from the responsible party to conduct the survey at the facility. Next, oral and written explanations were provided to the nurses and persons requiring care, who were referred to the responsible party, and consent was obtained. At that time, at the discretion of the facility authority, those who had severe cognitive impairment and could not understand the intent of the study were excluded from the study. If written consent was difficult to obtain due to physical paralysis, the situation was recorded in the field note and the person responsible at the facility wrote on behalf of the person requiring long‐term care. Personal information on persons requiring care was obtained from their medical records, and a paper‐based demographic survey was administered to the nurses. Nurses who participated in the study were given golden certificates as rewards. This study was approved by the Okayama University Graduate School of Medicine, Dentistry and Pharmaceutical Sciences and Okayama University Hospital, Ethics Committee (approval number: 2307‐023).

The target facilities were selected from nine facilities with an average size of 31 people or more in Okayama City among those that had applied for additional fees for functional training bathing, and were staffed with full‐time nurses using the Ministry of Health, Labour and Welfare's long‐term care service information disclosure system.

Study participants were full‐time nurses who belonged to the target facilities and had worked at the adult day service for at least 2 years, as well as persons requiring long‐term care who used both functional training and bathing services on the same day. Purposive sampling was used to recruit participants until the saturation of the analysis was confirmed. The purpose of the sampling was to recruit nurses who have heterogeneous characteristics and speak to the rich context of home care for older adults. Although it appeared that no new data had been extracted once the seven facilities had been surveyed through participation observation, two additional facilities were surveyed to confirm data saturation. Ultimately, 9 facilities, 10 nurses and 25 persons requiring nursing care became research collaborators; the characteristics of the target facilities and research participants are shown in Table 1.

**TABLE 1:** Overview of facilities and participants.

**Facilities (*n* = 9)**	
Occupations of people working in day care services	Occupational therapists, physical therapists, speech therapists, judo therapists, acupuncture and moxibustion therapists, Anma massage and shiatsu therapists, dental hygienists, registered dietitians, health exercise practice instructors, care support specialists, care workers, care helper Level 2 recipients, social welfare secretaries, office workers, unlicensed care workers, transportation drivers
Vital signs measurer	Nurses (7 facilities), caregivers (1 facility), undecided (1 facility)
Decision to bathe	1 Nurse (9 facilities)
Users per day	26–50
Bathers per day	13–32
Tank time	Within 5 min (6 facilities), within 10 min (1 facility), undecided (2 facilities)
Temperature in bathtub	38°C–42°C (8 facilities), no setting (1 facility)
Rest and relaxation environment	Beds (1–7), sofa set up, futon in tatami corner
**Nurses (*n* = 10)**	
Age	30–62 years (mean: 47.3 years)
Sex	Female (*n* = 10)
Qualification	3 assistant nurses, 7 nurses, 1 care support specialist, 1 public health nurse
Worked at day service	2–13 years (mean: 6.8 years)
Years of nursing experience	9–40 years (mean: 20.2 years)
Nurse practitioner duties	Welcoming and greeting patients; checking contact books; oral medications; eye drops; providing items necessary for wound care; providing fluids; toilet care; assistance with medications; handling falls, headaches, pain, and vomiting; checking the condition of oxygen equipment; indwelling bladder catheters; artificial bladder and artificial anus; exercise care; wound care; suction; enema; and stool removal
**Persons requiring long‐term care (*n* = 25)**	
Age	60–97 years old (mean: 79 years)
Sex	male: 11, female: 14
History of falls	yes (18), 1 with history of injury from bathing, 6 in wheelchairs
Living with family	yes (20) persons, living alone (3), living in senior housing (2)
Use of non‐day service	home nursing, short stay, home care, day care rehabilitation, home rehabilitation, home dental care, home drug management, welfare equipment, care cab, other day care services

*Note:* See Appendix B for diseases and symptoms of persons requiring long‐term care.

### 2.2 Overview of Adult Day Service Nurses' Decision‐Making Duties Regarding Bathing Patients

In the nine target facilities, nurses were responsible for several tasks (see Table 1), and vital signs were measured when a person requiring long‐term care visited the facility. In doing so, they not only measured quantitative values but also made decisions related to bathing by visiting a person in need of care and directly observing them while referring to reports from multiple professions.

### 2.3 Data Collection

The study consisted of participation observations, informal interviews (conducted on‐the‐spot with nurses during participation observation) and formal interviews (timed semi‐structured interviews in a private room using an interview guide based on Boyce (Boyce 2006), developed with a researcher familiar with qualitative research; see Appendix A for the interview guide).

Participation observations were conducted at each facility for 3 days between 8:00 a.m. and 5:30 p.m., with the time adjusted according to facility availability. Nurses were observed for a total of 110 h and 35 min, which included making bathing decisions for their patients. Formal interviews were conducted with nurses who were familiar with nursing care for older adults, after a preliminary trial of the interview guide to ensure clarity of the questions. Each interview lasted approximately 30–60 min and was conducted multiple times if the need for a break in the work or reviewing statements arose. Through formal interviews, which lasted 14 h and 8 min, we were able to gain insight into nurses' judgements. Questions such as ‘Why did you think that way?’ and ‘What is the reason for that?’ were used to clarify the background of their judgement.

The first author was responsible for all participation observations and interviews, and prepared field notes documenting the events observed, informal interviews during participation observations, reflections and interpretations. Formal interviews were recorded with permission and transcribed verbatim. Finally, 179,934 words (212 A4‐sized pages) were generated and retained as protected files. The participants were assigned a code and anonymised to prevent identification.

### 2.4 Data Analysis

Data were analysed using qualitative manifest and latent content analyses (Graneheim and Lundman 2004). The analysis was shared among the researchers using Excel for flexible code organisation and comparison. An example of this analytical process is presented in Tables 2 and 3.

**TABLE 2:** Examples of individual analysis.

Meaning unit	Condensed meaning unit	Sub‐category	Category
Prepare for deteriorating physical state affected by sleep conditions, living environment, as well as family rhythms and implement bathing	Bathe them while preparing for the deterioration of their physical condition during the bath due to sleep or family influences	Conduct bathing while considering the impact of the living environment on the activity	Bathing should be done while preparing for the effects of the living environment
I suspect that the physical condition is in terms of disease, but in my experience, I think it's more about the environment in which we live	In my experience, more physical problems are caused by the living environment than by the effects of the disease		
Older adults believe that their condition will improve with minor changes, such as their body not being able to keep up with changes in the environment, or the influence of the rhythm of family life	Focus on older adults' inability to cope with environmental changes and the impact of changes in family life rhythms		
Due to physical discomfort, hydration before and after bathing did not result in improvement; considering the stress caused by the noisy environment, the patient was placed in a quieter environment and the physical discomfort resolved	For those who are quiet at home, bathing in a noisy environment can be stressful and hence create a quiet environment for bathing	Bathe while paying as much attention as possible, even if the living conditions are not clear	
People with diabetes should consider food intake at home and avoid bathing at times before meals to prevent hypoglycaemia	Avoid bathing before mealtimes to reduce chances of low blood sugar		

**TABLE 3:** Examples of overall analysis.

Category	Subtheme	Theme
Bathing should be performed while preparing for the effects of the living environment	Preparing to deal with deterioration of physical condition caused by the home environment	Consideration of life at home
Keep in mind that the act of bathing, which is not done at home, can make you feel ill		
Be mindful of the possibility of physical deterioration due to a different environment than home		
Keep in mind the possibility of deterioration of older adults due to constipation and metabolic changes		
Considering the possibility of physical illness in those who are in the habit of not taking water at home, prepare water and a still room after bathing and carry out bathing		
If the family cannot take care of the older adult, take a shower or bathe for a short time even if there is a possibility of physical deterioration	Eliminating cessation of bathing due to lack of family caregiving skills	
Consider the family's strong desire to bathe, even if the person's requiring care has high blood pressure, if the blood pressure has decreased since the arrival and if their behaviour and complexion have not changed from normal, the patient is bathed		
Do not immediately stop bathing if the family or the patient strongly wishes to bathe, even if they are unsteady or unwell		
Bathe at adult day services as much as possible to reduce the burden on family members		
Bathe if there is no trauma, pain or change in movement, even if there has been a fall	Avoid discontinuation of bathing owing to familiarity with the characteristics of individual physical condition changes	
For those with physical functions that do not allow them to bathe at home, they should lie in bed when they are impaired and take a bath on the assumption that they will regain consciousness		
Bathe if there are no symptoms, even if there is weight gain due to heart failure		
Keep in mind that users of bathing services have reduced physical function and may lose consciousness		
Bathing to improve the skin condition of those who are obese and cannot bathe at home		
If you have oedema or are prone to hypotension, elevate the lower extremities after bathing		
Keep in mind that the environmental change that a person with cardiac disease undergoes during bathing may cause impaired consciousness		
Even after cramps and vomiting, he manages to bathe, because they cannot bathe at home		
Do not discontinue bathing even if there is a possibility that the persons receiving care at home may worsen		

First, in the individual analysis of each nursing staff member (Table 2), manifest content analysis was conducted to typify the semantic units and extract categories as concepts that explain the common features, using the following steps: (1) read the text repeatedly to understand it as a whole, (2) decompose the text into semantic units, (3) condense the semantic units, (4) group the condensed semantic units with common features into subcategories and (5) classify the text into categories.

Subsequently, an overall analysis was conducted. The overall analysis explored recurring elements across categories based on the categories extracted from each of the 10 nurses to identify the underlying meanings and structural aspects of their experiences. A latent content analysis (Table 3) was conducted to extract themes as concepts containing their latent meanings.

Subthemes were extracted based on category similarities, and themes were identified by increasing the level of abstraction based on the similarity of the subthemes.

During the entire analytical process, there was continuous discussion between the first author and a researcher familiar with qualitative research, and data saturation was confirmed. To finalise the theme, member checks were conducted with the nurses who collaborated in the study, and the themes were finalised under the supervision of a researcher familiar with home healthcare nursing.

## 3 Results

Analysis of the data resulted in 149 categories, with 13–16 categories extracted per nurse in the individual analysis. In the overall analysis, which combined these categories, 18 subthemes were extracted and 6 themes were identified (Table 4). Hereafter, themes are indicated by [ ], core semantic units are by “” and letters denote nurses A–J.

**TABLE 4:** Day service nurses' bathing decisions.

Theme	Subtheme
Gather information to compare with baseline	Observing from the perspective of whether the situation is different from usual
	Examining anomalies in measurements that consider the effects of individual diseases and internal medications
	Judgement based on subjective symptoms that can be complained of by the patient
Make observations based on information from others to understand the big picture	Confirmation based on multidisciplinary awareness
	Integrating information from multiple professions and family members with the caregiver's condition at the time of decision‐making
Give people time to get in shape	Maintaining physical condition prior to bathing and adjusting the environment
	Improvement of physical condition before bathing
	Choice of bathing method to prevent deterioration of physical condition
	Selecting bathing methods in accordance with functional decline in bathing‐related activities
Consideration of life at home	Preparing to deal with deterioration of physical condition caused by the home environment
	Eliminating cessation of bathing due to lack of family caregiving skills
	Avoid discontinuation of bathing due to familiarity with the characteristics of individual physical condition changes
Determining the need for medical institutions	Individual instructions obtained from the physician
	Determining the need for a medical examination
	Discontinuing bathing with speculation of possible acute hatred
Devise ways to communicate that promote collaboration	Information sharing among staff
	Multidisciplinary collaboration to minimise adverse effects of bathing
	Communicating information during day service use in preparation for the next bathing

### 3.1 [Gather Information to Compare With the Baseline]

This theme indicated a careful gathering of information on the differences between the normal condition of the person requiring care and the condition at the time of decision‐making. During participant observation and interviews, the nurses frequently identified differences from the usual conditions based on the individual criteria of each person requiring care rather than the facility's criteria, as shown by the following interview excerpts: ‘keep track of the usual condition and observe if there is any difference’ (E), ‘re‐measure accurately if any abnormality is felt’ (D) and ‘judge by individual standard values’ (B). By understanding the individual rather than the general standard values for each person requiring care on a daily basis for blood pressure and body temperature set by the facility, appropriate bathing decisions could be made.

### 3.2 [Make Observations Based on Information From Others to Get the Big Picture]

From this theme, we revealed that detailed observations were made based on information obtained from multiple professionals and family members, and that these observations were integrated to understand and determine the condition of the person requiring long‐term care, as shown by the following interview excerpts: ‘confirm with my own eyes based on information obtained from multiple professions and in advance’ (D) and ‘determine the bathing method after confirming with the person requiring care and family about the fall and observing the details’ (G). The nurses went to the person requiring care and observed their condition in detail, referring to information from multiple professions and family members. By comprehensively analysing the information obtained, they could accurately judge the situation of the person requiring long‐term care.

### 3.3 [Give Persons Time to Get in Shape]

Based on this theme, appropriate time was given to make decisions about physical conditions and environmental adjustments prior to bathing to ensure safe bathing as much as possible, as depicted by the following interview excerpts: ‘prevent physical condition by adjusting the environment and conducting systemic management such as supplemental food and medication according to the disease and age’ (J), ‘to prevent loss of consciousness during bathing due to insufficient fluid intake, always encourage fluid intake prior to bathing’ (A), ‘if the person requiring care is unable to straddle the bed due to physical weakness caused by lack of nutrition, and if it is presumed by observing their walking and functional training condition that the person requiring care is unable to do so, use shower baths’ (G). Nurses did not make judgements based solely on vital sign values immediately after the arrival of the person requiring care, but carefully observed the patient for a certain period of time, determined the optimal bathing method, managed the physical condition and adjusted the environment according to the individual's physical function and condition.

### 3.4 [Consideration of Life at Home]

This theme was observed in nurses performing bathing as much as possible during the day service, while considering the living conditions of the person requiring long‐term care at home, and after taking measures according to the individual physical condition of the person in need of care, as shown by the following interview excerpts: ‘If the family cannot take care of the older adult, take a shower or bathe for a short time even if there is a possibility of physical deterioration’ (A), ‘even after cramps and vomiting, he manages to bathe, because they cannot bathe at home’ (p. I) and ‘considering the possibility of physical illness in those who are in the habit of not taking water at home, prepare water and a still room after bathing and carry out bathing’ (B). While respecting the wishes of the person requiring care and their family members, the effects of bathing on patients' physical condition were estimated, and measures were taken according to individual characteristics before bathing.

### 3.5 [Determining the Need for Medical Institutions]

This theme indicated that nurses would work with physicians and healthcare providers to prevent acute exacerbations caused by bathing, as illustrated by the following interview excerpts: ‘if the pulse of a person requiring care is in the 40s and irregular, we consider it an emergency and discontinue bathing’ (p. I), ‘if the pulse value of the persons requiring care is not in line with the facility's standard, obtain instructions from the attending physician’ (E), ‘if the person requiring care has skin colour changes or abdominal discomfort, we encourage them to seek medical attention’ (F), ‘if the person requiring care has a fall, discontinue bathing and consider seeking medical attention’ (J) and ‘if the person requiring care vomits for unknown reasons, discontinue bathing’ (p. I). When nurses judged that it was difficult to improve the condition of persons requiring care simply by monitoring their progress, they decided to collaborate with an appropriate medical institution.

### 3.6 [Devise Ways to Communicate to Promote Collaboration]

This theme indicated the need to devise ways to communicate information to professionals and family members involved in bathing assistance so that persons requiring care could remain comfortable and clean, as shown by the following interview excerpts: ‘sharing information about bathing methods and home information among nursing staff and other professionals’ (J), ‘creating an atmosphere in which staff members can easily ask questions to each other and to other professionals to avoid problems with bathing decisions’ (A) and ‘explaining the intention of changing bathing methods to the person requiring care, family members, and care managers’ (p. I). By providing information in an easy‐to‐understand manner not only to each other but also to outside parties, such as family members and care managers, the staff prevented problems related to bathing.

## 4 Discussion

### 4.1 Characteristics of Adult Day Service Nurses' Bathing Decisions

We compared adult day service nurses' bathing decisions with concepts such as clinical judgement. Emphasis on comprehensive evaluation based on the experience and practical wisdom of (gathering information for comparison with the patient's baseline, considering their individuality) and the knowledge of (grasping the whole picture through observation based on information from others to make a decision) is common to existing clinical judgements (Connor et al. 2023; Johansen and O'Brien 2016; Manetti 2019).

In addition, a contemplative attitude is also required, rather than rushing to make a judgement (Connor et al. 2023; Manetti 2019), as in (giving the patients time to get in shape for safe bathing). Furthermore, a deep understanding of the person requiring care as a person living at home (Nishina et al. 2019), as in [deciding to bathe the person requiring care, considering their life at home], is another point common in existing clinical judgements. However, day service nurses' bathing decisions obtained in this study showed the following four characteristics.

First, information from multiple professionals, such as caregivers, rehabilitation workers and transportation drivers, was emphasised when making a comprehensive evaluation (e.g., [grasping the whole picture through observation based on information from others to make a decision]). As shown in Table 1, adult day services may have 25–50 people requiring long‐term care per day, and a single nurse may be responsible for making the decision to bathe the person requiring care. In addition, unlike decisions made at hospitals and other medical institutions, adult day services are facilities that are used only during the day and several times a week. Therefore, nurses need to make comprehensive assessments based on fragmented interactions. Therefore, awareness based on information from others is an important factor in judgements. In recent years, the need for multidisciplinary collaboration in community care has been highlighted (Asakawa et al. 2017; Bridges et al. 2011), and smooth communication is essential for good outcomes in recuperators. In adult day services, it is important to strengthen collaboration between multidisciplinary staff and family members to gather appropriate information.

The second characteristic was the understanding that the living conditions at home and the person requiring care's and their family's thoughts about bathing influenced the decision to bathe, as shown by [deciding to bathe the person requiring care, considering their life at home]. For example, the following factors should be considered: sleep disturbances may be caused by noise and the environment of family members living with the person requiring care. In addition, if the person requiring care is at home and always lying in bed, their physical condition may deteriorate because of sudden activities, such as transportation or functional training at adult day services.

Furthermore, there may be cases in which the recuperator and family members strongly desire bathing even if the medical condition of the person requiring care is unstable. In Japan's current super‐aged society, there is a need to shift from conventional ‘medical treatment to cure’ to ‘medical treatment to heal and support’ that prioritises maximising the patient's quality of life (Arai et al. 2015). Against this backdrop, nurses must make decisions that balance the wishes of those requiring bathing care with the need to ensure safe bathing while imagining the home life of people requiring care.

The third characteristic was unique in that it determined whether visiting a medical facility was necessary, as shown by [determining the need for medical institutions]. To date, no statistical data are available on emergency transport or consciousness disorders related to bathing during adult day services. However, in the [deciding to bathe a person requiring care, considering their life at home] theme, all nurses mentioned the experience of a person requiring care becoming unconscious during or after bathing [e.g., ‘bathing on the assumption that the person requiring care will lie down and regain consciousness when unconscious’ (p. I)].

Many individuals requiring care have multiple comorbidities (see Appendix B), and disease may be exacerbated. However, many such people requiring care are unable to visit hospitals and may not receive appropriate disease management. Furthermore, the risks of physical illness and loss of consciousness are latent in bathing practices in Japan (Nagasawa et al. 2001). In addition, older adults have multiple comorbidities, and individual differences are large, making it difficult to uniformly determine normal and reference values (Arai et al. 2015). Day services nurses measure vital signs such as blood pressure and body temperature before making the decision to bathe a person requiring care (Hayasaka et al. 2016). The results of this study show that nurses need to carefully observe not only quantitative values but also changes in symptoms (such as in walking, talking, reactions, facial expressions, grip strength, gaze and behaviour, heartbeat and skin colour; wounds that cannot be stopped; signs of falls; and vomiting of unknown origin) to determine whether bathing is appropriate.

To facilitate adults continuing to live at home, adult day service nurses, such as community health workers who serve as a bridge to medical institutions (Knowles et al. 2023), are required to manage health and, depending on symptoms, make decisions recommending medical visits. If the person requiring care can continue to bathe during the adult day service in cooperation with the medical institution based on appropriate decisions, hospitalisation or institutionalisation can be avoided and the person requiring care can maintain their life at home.

The fourth characteristic, [devising ways to communicate with others to promote collaboration], is important because adult day service workers come from diverse professional backgrounds, including social work, caregiving and rehabilitation, and have different qualifications, with some having none (Table 1). Consequently, there are individual differences in the knowledge of medical care, and ingenuity is required in the way information is communicated to work together with a common understanding. Specifically, it is considered important to communicate regularly, avoid using technical terms that are accepted only among medical personnel, and use specific and easy‐to‐understand expressions.

### 4.2 Limitations and Implications for Practice

The results of this study were obtained through focused ethnography of 10 nurses at nine facilities and, therefore, are not applicable to all adult day service bathing decisions. In addition, the information may have been biased because of the limited participation criteria for persons requiring long‐term care. Nevertheless, because of the detailed participant settings, the results could have been used in a similar setting. The data collection period for each site may have been inadequate, and extending the period may have yielded new results. Individuals from other countries residing in Japan who require care were not included in this study. As of 2024, there are 3,768,977 foreign residents in Japan, according to the Immigration Services Agency. Future research should consider including caregivers from diverse cultural backgrounds (Immigration Services Agency 2025).

Despite its limitations, this study clarified that the judgement of adult day service nurses in Japan was influenced by the unique bathing practices of people in Japan and the characteristics of the adult day service setting. We believe that we were able to capture the culture of nurse judgement in adult day services. Although the nurses' judgement must consider the living conditions and requests of the person receiving care, prioritising safety is essential to avoid serious conditions in the absence of physicians during the adult day service. Adult day service nurses are required to make decisions while balancing the wishes of the person requiring care and family members with the risks associated with bathing. Making decisions amid a busy work schedule entails the possibility of overlooking the wishes of those requiring care and the risk of sudden changes. Therefore, it is important to establish a foundation that allows nurses to consolidate their awareness of multiple professions and information from family members. For instance, it is important to develop a system that allows for communication among various professions regarding the non‐quantitative judgement factors valued by nurses, thereby facilitating decision‐making that aligns with the needs of the individual receiving care by creating relevant indicators and holding study sessions.

## 5 Conclusion

Six themes regarding bathing decisions by adult day service nurses were identified in this study. Adult day service nurses made bathing decisions for persons requiring nursing care by focusing on information provided by others. The uniqueness of this approach was recognised in the way it considered home life and wishes for bathing, as well as the way it determined the need to connect to a medical institution and communicate this to family members and other professionals. By building an infrastructure that can aggregate information from multidisciplinary awareness and family members, adult day service nurses may be better able to facilitate decisions that are in the best interests of caregivers in need.

## Author Contributions

All authors contributed to the conception and design of the study, data collection, analysis and interpretation were involved in the preparation of the paper and reviewed the final manuscript.

## Ethics Statement

This study was approved by the Okayama University Graduate School of Medicine, Dentistry and Pharmaceutical Sciences and Okayama University Hospital, Ethics Committee (approval number: 2307‐023).

## Consent

Written informed consent was obtained from the responsible persons at all study sites and from the participants after an oral explanation.

## Conflicts of Interest

The authors declare no conflicts of interest.

## Data Availability

The data that support the findings of this study are available from the corresponding author upon reasonable request.

## Appendix A Interview Guides

1. How did you come to work at the day care service?

*This question is not directly related to the content of the study, but it is a trigger question to make the interview more relaxed and facilitate eliciting nurses' thoughts.

2. Is there anything else that is part of a day's work by the nurses other than what you have seen?

3. Regarding whether to assist with bathing as scheduled, the following questions were asked in the course of the conversation:

- Can you describe the details of what you do with special attention, such as regarding decision‐making?
- What kind of information do you need if you decide to shorten or discontinue the programme?
- Who do you consult with when you are unsure about making decisions regarding?
- How do you evaluate the decision you have made?
- What are some examples of cases in which your judgement was inaccurate?
- What, if any, decisions were made after you felt your judgement was inaccurate?

4. Do you have any other concerns about whether or not to provide bathing assistance as scheduled, other than the patient's information?

5. Regarding what the nurse paid attention to in order to ensure that the decision is communicated correctly, the following questions were asked:

- What do you consider important when communicating your judgement to social workers and caregivers?

6. How do you resolve differences in judgement with social workers, caregivers and other people involved, such as family members?

7. What do you think is the value of your judgement?

8. What do you think are the benefits that the users and their families get?

9. Last but not least, what kind of education do you think would benefit inexperienced day service nursing staff?

## Appendix B Diseases and Symptoms of Persons Requiring Long‐Term Care

**Table:**

Person	Age	Diseases and symptoms
a	80s	Hypertension, dementia, diabetes, bone fracture (multiple times)
b	80s	Ulcerative colitis, heart disease
c	70s	Bone fracture (lumbar)
d	90s	Knee osteoarthritis
e	70s	Multiple system atrophy, bone fracture (lumbar), hypertension, orthostatic hypotension
f	70s	Stroke, epilepsy, bone fracture (lumbar)
g	80s	Diabetes, stroke, heart disease
h	70s	Hypoglycaemia symptoms, bone fracture (upper limb)
i	90s	Mammary cancer, hypertension, hyperlipidaemia, esophagitis, autonomic nervous disorder, skin damage, knee osteoarthritis
j	70s	Stroke, abdominal aortic aneurysm (after the operation), heart disease
k	90s	Dementia, insomnia, hypertension, colon cancer, stroke, aspiration pneumonia, osteoporosis, bone fracture (multiple times)
l	90s	Bone fracture (thoracic and lumbar), heart disease, osteoporosis, chronic hepatitis
m	90s	Lung disease, stroke, disuse syndrome, hypertension, heart disease, anxiety, dementia, osteonecrosis
n	80s	Arthritis, lumber spondysis, knee osteoarthritis, hyperlipidaemia, vertigo
o	80s	Parkinson's disease, stroke, anaemia, bone fracture (femur)
p	60s	Stroke (dysphagia and dysarthria present)
q	70s	Stroke (dysphagia and dysarthria present)
r	70s	Stroke (pulmonary aspiration), disuse syndrome
s	80s	Dementia, adjustment disorder, bladder cancer, Parkinson's disease, hypertension
t	70s	Stroke, asthma, hypertension, morbid obesity, knee osteoarthritis
u	70s	Stroke, gastric ulcer
v	60s	Articular rheumatism
w	80s	Stroke, diabetes, enlarged prostate, hypertension, lumbar spondylosis, knee osteoarthritis
x	60s	Stroke, hypertension, hyperlipidaemia, constipation, esophagitis
y	80s	Lumbar spinal canal stenosis, chronic kidney disease, cervical cord injury, cataract, glaucoma, urinary retention
